# From Disruption to Reconstruction: Implementing Peer Support in Homelessness During Times of Crisis for Health and Social Care Services

**DOI:** 10.5334/ijic.8594

**Published:** 2025-01-16

**Authors:** Mathieu Isabel, Daniel Turgeon, Émilie Lessard, Andreea-Cătălina Panaite, Gwenvaël Ballu, Odile-Anne Desroches, Ghislaine Rouly, Antoine Boivin

**Affiliations:** 1 Department of Family Medicine and Emergency Medicine, Université de Montréal, Canada; 2 Dalla Lana School of Public Health, University of Toronto, Canada; 3 Canada Research Chair in Partnership with Patients and Communities, CHUM Research Center, Canada; 4 School of Public Health, Université de Montréal, Canada

**Keywords:** peer support, homelessness, primary care, community care, implementation, crisis

## Abstract

**Introduction:**

Peer support workers—people with a significant lived and living experience of a social or health condition—use their experiential knowledge and obtain training to help and care for others. They are integrated in different clinical settings, including those for people experiencing homelessness. Most research on peer support implementation in homelessness has not considered the *timing* of the implementation, particularly in periods of crisis.

**Description:**

During the COVID-19 pandemic crisis, a participatory research project examined the integration of a peer support worker in a primary and community care clinic that serves people experiencing homelessness in Montreal (Canada). This article presents a narrative case study analysis of the specific data on implementation derived from this project.

**Results:**

Three main learning points are of interest regarding implementation: 1) crises can precipitate challenges but also particular opportunities for the implementation of peer support initiatives in homelessness; 2) even during a crisis, certain key steps cannot be skipped when the goal is a successful implementation; and 3) research can be an external asset for clinical teams as they struggle to deliver care during periods of crisis.

**Conclusion:**

Peer support initiatives in homelessness can be implemented in the Canadian context during periods of crisis—for example, the COVID-19 pandemic—for health and social care services. Moreover, the concept of *crisis* itself can be reexamined by clinical and research teams worldwide as potentially enabling the implementation of novel initiatives.

## Introduction

*“[The peer support worker] came at the right moment into the [clinical] team. We were in a period where we were facing a general loss of meaning.”*

*“We were motivated to welcome him because it was an innovative project that brought us back to what we really wanted to do as a team, back to our mission. During this period of uncertainty [of the COVID-19 pandemic], he really helped us.”*

*Clinical team member, Focus group*

In Canada, at least 235,000 people experience homelessness every year [1]. They are confronted with numerous intersecting psychosocial and medical challenges, including poverty, trauma, and a lack of access to affordable housing [2]. They also present different physical and mental health conditions, both acute and chronic [3]. As a population, people experiencing homelessness present with both premature aging and mortality [4,5]. Many of them may avoid health care based on a mistrust of the health-care system, a difficult access to it, or previous discriminatory experiences from providers [6].

Peer support workers—people with a significant lived and living experience of a social or health condition—use their experiential knowledge^1^ and obtain training to help and care for others [7,8]. Overall, the integration of peer support workers in clinical settings has been associated with a better quality of life for the people benefiting from these services [9], and an easier navigation of the health and social services system [10]. Since the mid-1980s, several peer support programs have been developed worldwide for mental health [11] and harm reduction for drug use [12]. Many of these peer initiatives have stemmed from the need to reach so-called “vulnerable populations” in the contexts of public health crises, such as adapting the distribution of harm reduction materials (condoms, injection kits) with the aim of reducing the spread of HIV [13] and hepatitis C [14].

Particularly, peer support workers in homelessness are seen as “experts by experience” [8], individuals who have experienced homelessness in the past and who have enough stability in their current residential and personal life to accompany people currently experiencing homelessness. Concretely, these peer support workers can help with housing searches or the navigation of the health and social services system, as well as offering active listening and support. Different peer support initiatives for people experiencing homelessness, both in clinical and community settings, have demonstrated positive results in various countries, including an improved housing stability, an increased access to and trust of primary care services, and a significant improvement regarding mental health and substance use [15,16,17,18,19]. Many clinical guidelines addressing interventions with people experiencing homelessness are now recommending the integration of peer support workers within clinical teams [20,21].

Different facilitators and challenges have been identified regarding the implementation of peer support initiatives in homelessness, which include 1) the importance of considering institutional, professional, and practical barriers to implementation [22]; 2) the provision of the peer support worker with a clear role definition and support system [23,24]; and 3) the need for co-building and adapting the intervention and its evaluation with respect to the peer support workers, community partners, and people experiencing homelessness [25].

To date, most research on peer support implementation has focused mainly on individual, collective, and organizational factors that affect the implementation process. However, the *timing* of the implementation rarely has been studied. As the first wave of COVID-19 hit the world in 2020, the access of people experiencing homelessness to clinical and community resources were severely restricted both in Canada and worldwide, and so services needed to be adapted [26,27,28]. In this context, we wondered about the feasibility of integrating a peer support worker in times of crisis, particularly in a clinical team offering services to people experiencing homelessness. More specifically, we wondered whether social and health crises offer favorable windows of opportunities or specific challenges for peer support implementation?

The concept of *crisis* itself needs to be clarified before we go further. Crises—whatever form they take—disrupt the regular functioning of individuals and communities. Regarding the present project, the COVID-19 pandemic represented such a major crisis in which public health and social inequalities intersected with one another [29]. This pandemic put an unprecedented strain on people experiencing homelessness, community organizations, and the health care system and its workers. Therefore, we will not refer to the COVID-19 crisis as affecting a number of particular individuals, but rather as a societal level crisis that heavily impacted health and social care delivery. Of particular importance, we will not present the COVID-19 pandemic as a static crisis that simply served as a contextual element for this project. Instead, we will present the COVID-19 crisis as a dynamic one that heavily influenced the implementation of this project.

Thus, we distinguish between the pandemic crisis and a project-related crises. In fact, our project itself went through different stages of crises, each of which presented unique opportunities and specific challenges that influenced its implementation in homelessness. As we will explain further later, although these project crisis stages closely followed the COVID-19 epidemiological pandemic waves, the stages were not necessarily *directly related* to the waves.

Therefore, our study seeks to inform practitioners, managers, and researchers on the *how* and also the *when* of peer support implementation timing and strategies in homelessness. Our objectives are to: 1) describe this peer support initiative; 2) analyze its implementation trajectory in a crisis context, from the co-design with community and clinical partners to institutionalization; and 3) reflect on the timing of peer support implementation during the various stages of a crisis, including challenges and opportunities.

## Description of the practice

### Situating the peer support initiative

Before going further, we need to clarify an important point. This project was not developed with an implementation research orientation in mind. It was designed first to address clinical, community, and human needs during a period of crisis. For people experiencing homelessness, tackling the COVID-19 crisis did not simply equate to avoiding and containing the virus. In Montreal, clinical services in primary care settings were reduced significantly to favor acute in-hospital care. Shelters greatly reduced their bed capacity, and community organizations diminished their services. This situation required an integrated response, both medical and social, to the unique challenges of being homeless during an unprecedented event. Thus, our peer support initiative was an attempt to provide such a response using the building blocks from a previous research-action project. Then, from this experience, the research team gained valuable knowledge about what it means to implement peer support initiatives in times of crisis. This article is a written account of these lessons.

One of the goals of this research project was to integrate a peer support worker in homelessness in a primary and community care clinic in Montreal (Quebec, Canada) during a period of crisis (namely the COVID-19 pandemic). Another goal was to evaluate the effects of this initiative. Thus, this article focuses particularly on the implementation process of the peer initiative and explores how its timing, during a pandemic, helped or hindered its implementation. Findings regarding the particular effects of the peer support worker in a context of homelessness are addressed in another publication [30].

Our project was based on a participatory research approach [31,32] that engages peers and healthcare teams as partners in research [31,33]. Participatory research helps to transform the tacit knowledge of peers and healthcare teams into explicit knowledge that can be shared in a tangible form [34]. Our project design was based on an existing model of peer support in community care (Caring Community). Caring Community is a participatory research program that positions peer support as a bridge between health and community care [35]. Established in Montreal in 2016, it was founded by a patient (GR) and a family physician (AB), thus combining experiential and scientific knowledge. Caring Community explicitly adopts an individual strength-based approach while offering a community-wide perspective on health. It brings together peers with a diversity of lived experience and connects them with a broad range of local health and community resources (primary care, mental health, aging, end-of-life). Since the Caring Community model—originally designed and piloted in a community-based primary care setting—is characterized by support for people with complex health and social issues, our project adapted it to the reality of homelessness.

As a research site, we chose a primary and community care clinic offering services to people experiencing homelessness in downtown Montreal. For decades, this clinic has been offering integrated medical and social care for people experiencing homelessness. The clinical team is composed of social workers, nurses, physicians, psychosocial educators, and clinical counselors. Over time, it has developed collaborative outreach programs with different street workers, community organizations and major shelters in Montreal, as well as community-based initiatives with the police service.

As part of this participatory research project, a peer support worker was integrated in this primary and community care clinic. As we will explore later, the COVID-19 pandemic already had begun to affect healthcare clinics and community organizations in Montreal when the peer support worker was integrated. Social distancing was in effect, clinical services for patients were drastically reduced, and community organizations and shelters had limited resources to offer. Nevertheless, even during this challenging time, the peer support worker was able to do his work. He offered support through shared experiences, role modeling, and linkages to health and community resources; and he collaborated with health professionals, community workers, and our research team. The range of his interventions was vast even in the midst of a global pandemic: helping people experiencing homelessness with housing searches, accompanying them to appointments or court hearings, supporting them through difficult situations and the consequences of the pandemic, and so on. The peer support worker intervened in the clinic, community organizations and shelters, and directly in the street.

### The research team

The research team benefited from the diverse collective experiential and academic knowledge of all its members, who represented a mix of “insider” and “outsider” perspectives. It included a mid-career clinician-scientist with 20 years of experience in patient and community engagement science (AB), the lead peer support worker from the Caring Community program with more than 50 years of experience in peer support and patient partnership (GR), a researcher involved with the literature review and data collection (GB), a post-doctoral researcher specialized in developmental evaluation, and a family physician and medical director of the homelessness clinic that hosted our initiative (MI). Later on, other members joined the research team including the peer support worker of this project when he was hired (DT), a researcher specialized in participatory and population health research (EL), and researchers specialized in peer support research (AP, OAD).

Throughout the implementation process, the research team built a diverse coalition of partners to support the peer support worker in his integration. In fact, he was connected with a well-established peer-led community group and experienced peer support mentors. Presentations were offered to the clinical team before and after his arrival regarding the best practices in peer support. A learning circle also was organized to share common experiences from similar projects in peer initiatives (not necessarily related to homelessness) and to learn from one another.

### Documenting the implementation trajectory

Our methodology for this article draws on empirical observations/learnings derived from this participatory research project using a narrative case study approach [36,37]. For this project, we used a large series of data collection tools to document the perceived effects of the peer support worker’s presence on clinicians, people experiencing homelessness, community partners, and the peer himself [30]. However, to produce this article, we used a subset of data on implementation gathered by using certain tools: 1) a logbook to track all the significant steps of the implementation trajectory, including perceived barriers and facilitators (collected by DT, GB, ACP, M); 2) notes from meetings between all members of the research team (n = 38; on average bi-monthly); 3) bi-monthly debriefing sessions between the peer support worker (DT) and a member of the research team; and 4) a focus group with clinicians during which specific questions about implementation were asked. Our analysis is structured chronologically around the first three waves of COVID-19 and beyond, from July 2020 to December 2021. We used data from the logbook and the focus group as well as notes from the research team’s meetings and the debriefing sessions to produce a narrative description of the integration of a peer support worker in a primary care team during a time of crisis.

## An implementation trajectory through different stages of a crisis

The implementation of this peer support project evolved through three stages of crisis. The first occurred during the first wave of COVID-19 in Montreal (spring 2020). This created an opportunity to position the building blocks of the peer support initiative. A grant was awarded, the research initiative was designed, and the peer support worker was hired. The second stage unfolded during the following waves of COVID-19 (autumn 2020 and winter 2021) when people experiencing homelessness were severely affected by the virus, and clinical teams and community organizations were greatly impacted as well. During this stage, the peer support worker was integrated in the clinical team, and data was collected. The third stage of the crisis occurred during the spring of 2021. Although the COVID-19 cases were going down, the initiative went through another form of crisis. Following a grant refusal, the project experienced a period of uncertainty that finally led to financial and institutional sustainability.

### Crisis stage #1. COVID-19 hits: Paving the way for peer support

This first stage of the crisis coincided with the initial COVID-19 wave in Montreal in the spring of 2020. At that time, non-essential in-person services at the clinic as well as outreach activities in the street and community organizations were significantly reduced, which limited clinical resources for people experiencing homelessness. Masking protocols and other protection/distancing measures also were in effect, making interventions particularly challenging. In this pandemic context, policy makers and funders were seeking rapid responses and solutions regarding how to address the consequences and uncertainty of COVID-19 in different populations. This situation led to the creation of opportunities for funding and innovations that focused on building partnerships at various levels, whether in community or clinical settings. Our project was funded initially by one of these grants. Although, at that time, the general population was beginning to be infected by the virus, people experiencing homelessness were relatively spared from the initial major outbreaks. Various explanations were offered for this phenomenon, including the beginning of the spring season when people experiencing homelessness spent more time outside and slept in places other than crowded shelters. This initial crisis context motivated the research team into action, and unexpectedly provided the time for a better co-designing and co-creating of this project.

Based on the literature and previous experiences with the Caring Community initiative, the research team was mindful to create and maintain the best conditions under which such a project could develop. From the initial phases, the team was active in meeting and building partnerships with local clinicians, managers, and community collaborators already involved in other peer projects. During the first four months of the project (from July 2020 to October 2020), the research team had more than 16 meetings (~30 hours) and another 25 meetings with local clinical and community partners and senior peer support mentors. They also met with key informants who suggested who the team should talk to next, which included representatives of a local not-for-profit organization that supports the training, recruitment, and integration of peer support workers in clinics. The research team was interested in hearing about their experiences concerning the implementation of a peer support initiative, what they considered as the fundamental components, and what they would have done differently.

As mentioned earlier, the research site was chosen because of its demonstrated ideological, professional, and institutional support. The lead physician of this primary and community care clinic (MI) became part of the research team. A few meetings were arranged initially with high-level and local managers of the clinic to present the team’s ideas and to listen to their vision about such a project and ensure their institutional support would be offered. They had attempted to implement such an initiative a few years previously but due to logistical issues were unable to be successful. Thus, these managers were quickly on board and ensured that the peer support worker would benefit from the same administrative support as the other members of the team (e.g., designated office space, ID card, computer access). Also, two preparation meetings were arranged with the clinical team of physicians, social workers, and nurses to introduce the peer- support worker’s role and identify any potential barriers and facilitators to the implementation of such an initiative. During these preliminary discussions, most clinicians, as well as some community partners working with them, expressed an eagerness to integrate peer support workers within their practice. Nevertheless, some clinicians were initially reluctant to integrate a peer support worker within their team based on her/his potential impact on their practice or concerns about the peer support worker’s level of “stability” and whether he/she needed to be “protected.” However, for the majority, peer support “fitted” with their shared mandates around client autonomy, psychosocial support, and rehabilitation.

After these essential preparatory steps, the research team was ready to recruit the peer support worker to join the project. At this stage, a significant amount of time had been taken to ensure the ground was fertile to deploy the initiative, although the finer details of the peer support program had not yet been decided. Regarding the launch of the project, the peer support worker was the essential missing member of the team. Following the identification of potential candidates through peer support organizations met with during the consultation period, the team held interviews and hired a formally trained peer support worker to officially join the research team in November 2020.

Two social workers from the primary and community care clinic with previous experience working with peers also were identified as references to facilitate the integration process of the peer support worker. They provided him with an initial orientation at the clinic, introduced him to community workers and potential service users, and acted as easily accessible reference supports for him throughout the project. These two social workers also were authorized by the clinic manager to attend research meetings when necessary, as an in-kind contribution.

The peer’s integration in the clinical team while also being part of the research team involved a series of clarifications. The research team reinforced the importance of clearly defining the peer’s role with managers and local health authorities. Although in the initial phases of the project the peer support worker quickly was recognized as a full member of the clinical team, he was initially integrated as a partner rather than an employee of the healthcare institution. While the peer support worker had access to the clinical team offices and team meetings, we worked with confidential peer support intervention notes that were distinct from the medical files. This approach enabled the sharing of relevant information to facilitate co-interventions while at the same time protecting any confidential information that clients may only want to share with the peer support worker or health professionals, as long as the safety of the clients or others were not at stake.

At the end of this first crisis stage, the building blocks were in place, the peer support worker was hired, and the clinical and research teams were ready and on-board. The peer support intervention initiative was about to deploy, and then the COVID-19 cases rose again.

### Crisis stage #2. COVID dismantles services: Peer support as a welcomed ally

The second crisis stage in this project coincided with the second COVID-19 wave in Quebec in December 2020. This time, people experiencing homelessness were hit harder than during the first wave. COVID-19 cases were exponentially accelerated with large outbreaks and high death rates in the homeless populations. State enforced curfew measures were imposed during the cold winter months (following protests, these measures later would be canceled for people experiencing homelessness), and many community shelters drastically reduced their services. Of particular importance, professionals in various primary and community care teams (including ours) were reassigned to hospitals and other COVID-19 dedicated units. In the context of staff shortages, added to already strict infection-prevention protocols, only essential services were maintained at the community care clinic we had chosen for our project. So-called “regular” services, including many social, nursing, and medical services were suspended for many of the people experiencing homelessness who usually attended the clinic on a more “regular” basis. Similarly, the research team was confined to work remotely from home.

In the middle of this crisis, the health and social care system turned suddenly to a “whatever works” mode, and innovative solutions were welcome. As evidenced by the data from our focus groups and interviews, the peer support worker and research team were favorably received. Although many health professionals from the clinic had to reduce their services, a majority of the activities with the peer support worker were maintained because he was an addition to services, somehow fitting in between the clinical team and the research team. His work schedule was designed based on a two days a week presence at the clinic. This small-scale approach gave the peer and the clinical team time to adjust without being overloaded. Although the peer support worker was paired initially with the remaining healthcare professionals at the clinic in the initial weeks of his mandate, he also began independent work and was quickly recognized by community organizations as a unique contributor to supporting people experiencing homelessness during this crisis period. The peer support worker continued regular on-site visits at three important local community organizations serving the homeless population. To facilitate linkages with potential clients, one community organization even posted a description of the peer support workers’ role.

In the midst of this stage of the public health crisis, the research team as a whole remained present. Virtual research meetings were held every two weeks to obtain a very accurate and up-to-date report of the situation on the ground. Thus, communication channels were kept open between the clinical team and research team throughout the crisis. Although the whole research team was not physically present at the clinic, the clinical team mentioned how they felt supported by them. Clinicians and administrators also felt that the peer support initiative was well taken care of by the research team during this crucial implementation period. As a matter of fact, the clinical team expressed that, in a rare turnabout, research was at the service of clinical work rather than vice versa.

### Crisis stage #3. The end of innovative short-term solutions: From funding rejection to sustainability

Although it is difficult to conceive that a grant refusal could be a blessing in disguise, for this project, it was. The third stage of crisis in this project—certainly different from the two previous ones—began when COVID-19 cases were starting to decline in the spring of 2021. Philanthropic foundations that sought short-term innovations to address the immediate consequences of the pandemic were now pivoting to longer term objectives and different funding priorities. Thus, our project renewal application was declined, and our start-up funding ended.

People involved in our project quickly tried to initiate other funding strategies, since rapid funding alternatives seemed impossible. Those who had first-hand experience of this peer support initiative in homelessness were more inclined to appreciate its added value, beyond the short-term context of the pandemic. First, the peer support worker expressed a desire to continue his work. Then, given the project’s alignment with its strategic orientations, the lead researcher was determined to continue the initiative and decided to use discretionary funds to temporarily cover costs. Although this meant that planned developments were not possible in the short term, the peer support worker’s place in the research team was secured, at least temporarily.

The funder’s refusal also made the clinical team acutely aware of the relevance of the peer support initiative and the loss it would represent should it come to an end. As mentioned in our implementation logbook, a few weeks following the end of research funding, an executive manager from the primary and community care clinic contacted the research team. For her, it seemed impossible to abandon this initiative. After only a few months, the peer support worker had been welcomed by other members of the team, and he had been recognized as an asset by a variety of community organizations. The executive manager already had begun to explore options to permanently integrate the peer support worker as an employee. Difficulties still needed to be overcome, such as the lack of a job title for a peer support worker in a highly bureaucratic organization, but the desire was evident. With the leadership of this manager and the keen support of the clinical team, the peer support worker was officially employed two months later by the local health authority and the clinic.

The third stage of the crisis generated a lot of uncertainty, both in terms of finance and perennity. However, it also created the necessary conditions to raise this peer support project in homelessness to the next level. Thus, this initiative went from a pilot-project under the leadership of a research team with short term philanthropic funding to a secured position within a clinical team funded through a recurrent state budget for health care and social services. Beyond a job title, this success represented an important milestone in recognizing a professional occupation based on lived and living experience within the health care system in Quebec.

## Discussion: The Perfect Storm?

Data on implementation derived from our participatory research project showed that peer support initiatives in homelessness can be implemented during periods of crisis such as the COVID-19 pandemic. Three main learning points are particularly of interest with respect to this project: 1) crises can bring on numerous challenges but also particular opportunities for the implementation of peer support initiatives; 2) even during a crisis, certain key steps cannot be skipped when the goal is a successful implementation of a peer support program; and 3) research can be an external asset and an opportunity to breathe, innovate, and adapt while a clinical team struggles to deliver care and continue day-to-day activities.

### Crises: Unique opportunities and difficult challenges

The COVID-19 crisis and the various crisis stages of this project offered different opportunities for innovation. This initiative was implemented due to a synergic combination of many elements. The first was a clinical team with decades of experience in community-based care, a solid tradition of collaborating with community organizations, and a strong desire to integrate peer support workers within their team for the first time. The second element was a diverse research team with experience and expertise in implementing various peer intervention initiatives. The third was a series of community partners who valued the role that peer support workers can play in the ecosystem of community care. The final element was a peer support worker who had a unique combination of life and professional experience, training, and capacities; and a desire to work on such a pilot project.

This is not to say that all these conditions must be present to successfully implement a peer support initiative for people experiencing homelessness. Rather, our data show that crises can remove certain barriers and serve as innovation producing incubators when unique and local factors are identified as resources and brought together. Implementing a peer support initiative in homelessness during a period of crisis goes beyond the application of implementation theories. Indeed, it requires the materialization of concepts, principles, and values into people, relationships, and tangible practices. The recruitment of the peer support worker is an important example of this. In such a “pilot” initiative, the recruitment process almost becomes an art. It is a fundamental task that involves the entire research team in recognizing unique opportunities and who is the right person currently in a favorable position in his or her life journey to become a peer support worker. It is about finding a person who is still sufficiently connected to his or her experience of homelessness while being able to integrate into a clinical team.

Obviously, during this project, some barriers existed to implementation. As previously discussed, some clinicians were initially concerned about the integration of the peer support worker, some managers were preoccupied with his access to medical files and confidentiality, and an official job title to “fit” him into did not exist. The COVID-19 crisis also put a particular strain on the peer support worker regarding the pressure to deliver services in this context as well as security concerns relating to his exposure to the coronavirus. Nonetheless, our project showed that a crisis context can remove, or at least flatten, some of these implementation obstacles. The health care system as a whole was willing to find alternative solutions for confronting this crisis, long administrative processes often were removed, and clinical teams and managers were willing to embark on projects that “made sense.”

### Going “at the speed of trust”: The necessity of relationship-building, collaboration, and team support

Even in a crisis situation, certain important steps cannot be skipped when implementing initiatives. In Emergent Strategy [38], actor and activist adrienne maree brown mentions how social change needs to be anchored in meaningful relationships that “move at the speed of trust.” Thus, we argue that to make participatory research ethical and sustainable over time, one must go at that exact same “speed of trust.”

The many hours initially spent in meetings with stakeholders were fundamental for this project. The research team took the time to assess the readiness for this innovation, create relationships, develop trust with local partners, and adapt strategy to the changing conditions of a period of crisis. Similar to Ibrahim’s [22] views on the implementation of peer support initiatives, the research team reinforced the importance of considering institutional, professional, and practical barriers to the implementation of peer support programs, such as finding an appropriate fit with a given organizational structure. These numerous preparatory meetings also enabled the team to explore reluctance because some clinicians and managers had valid concerns about the implementation and some were not that interested in collaborating with a peer support worker. Before hiring the peer support worker, the research team was able to address these concerns.

This process definitely was time consuming. However, even in a crisis situation when rapid actions often are needed, numerous benefits can be gained from resisting the urge to implement an action at all costs and from forcing a team to slow down while offering pragmatic solutions in real time. The implementation process became as important as the implementation per-se. The relationship building period during the first stage of the crisis enabled us to better confront the second and third stages. Haldane [39] has described how, during the COVID-19 pandemic, countries who responded most efficiently to the crisis showed high levels of collaboration and coordination between all sectors of society involved in the pandemic response. Our project chose this collaboration approach during the periods of the crisis at a smaller level. The constant communication between the research team and the clinical team throughout the crisis as well as the frequent physical presence of members of the research team at the clinic helped to cultivate mutual trust and respect.

Progressively, the peer support worker became known by the professionals working at the clinic, community organizations workers, and people experiencing homelessness with whom he met in both clinical and community settings. In the vast majority of places he worked, his unique role and expertise were better understood, and he was recognized as an expert and colleague by most people. His presence—and the unique role he embodied—became the thread that connected clinicians, community workers, health authorities, people experiencing homelessness, and researchers.

### Research as an external asset for clinical teams during periods of crisis

Can research teams be useful during periods of crises? Can they be present without being “in the way” when concrete and rapid actions are needed? These were the sorts of questions that the research team constantly asked itself. And based on data on implementation coming from of our participatory research project, the answer is yes.

The strong collaboration between the clinical and research teams served as an important element of the implementation process during a period of crisis. From day one, the research team included the head physician of the clinical team (MI) who served as a facilitator between the two teams. He was able to report and contextualize various elements that were important to the research team while communicating quickly with clinicians, managers, and local health authorities. Later on, other clinicians were involved in the research team meetings and authorized to do so by their manager as an in-kind contribution because that manager firmly believed in the importance of the project and the necessity to keep the communication channels open. Throughout the project, members of the research team met with community organizations and clinicians to conduct interviews or focus groups with them or with people experiencing homelessness, communicated research findings during team meetings, and provided brief updates about the evolution of the implementation process during shorter meetings.

Nevertheless, even with this favorable context, it was important that the participatory research team did not come to this project “empty handed.” The research team was welcomed because it brought both leadership and resources, and of particular importance, it took the initial leadership role. Although the clinic already was convinced of the importance and potential value of a peer support worker, the COVID-19 crisis put a high strain on the clinical and community teams, which made it difficult for them to lead new implementations of practice innovations, build partnerships, support recruitment and role clarification, and ensure ethical and institutional oversight. Therefore, the research team leadership during the early phase of the project was a valuable practical support and a key facilitator in the initial experimentation with the peer support worker. This leadership role often has been described as a reassuring presence by clinicians and managers, and it helped to clarify that research could be adapted to the reality and the needs of the clinical world in its response to the COVID-19 crisis.

The funding crisis created a lot of uncertainty, but this situation was far from being unique to our project. Many health and social care services around the world often are confronted with funding cuts or frequent optimization processes [40,41]. In Canada, as in other countries, it is not rare that pilot project initiatives are not funded and scaled up in the long-run [42,43], which makes the process of even starting pilot projects such as ours both stimulating and risky. Although we gathered the right people around our project, certainly chance and timing were in play to bring us a high level manager in the healthcare system who believed in our project and was willing to open her budget purse. This success probably was related to first choosing a site where the soil was fertile to begin with and in which participants had the best prospect of aligning their visions.

The transition in funding during the third stage of crisis of this project also triggered a transition in leadership. Following the clinic’s official hiring of the peer support worker, the leadership role played by the research team changed from being in the foreground, providing reassurance and support to the clinical partners (in addition to its role in research), to a more background role of support for the clinical team. Nevertheless, it appeared important to the research team and the peer support worker to still be paid partially by the research funding for the specific time devoted to research and knowledge transfer activities. It enabled him to continue his researcher role in developing partnerships and in maintaining a presence in learning circles where he shared his experience with other peers, so they could continue to learn from one another.

### Limitations and further studies

This article explored the implementation trajectory of a peer support initiative in homelessness mainly from the perspectives of a peer support worker and members of a clinical team. Thus, it does not represent the entirety of the voices involved in this project, but rather sheds light on the critical elements involved in such an implementation trajectory through the experiences of the peer support worker and members of the clinical team. The implementation-specific elements referred to also were part of a larger data collection process that was used to evaluate the perceived effects of the peer support program. Although these data on effectiveness (obtained from a series of semi-structured interviews and focus groups) were not used specifically for this current article, they were analyzed nonetheless to explore the perceived effects of the peer support program on people experiencing homelessness, clinicians, administrators, and community partners. It could be interesting to conduct another study documenting the implementation trajectory of such a program from the perspectives of all these various actors.

## Conclusion

Peer support initiatives in homelessness can be implemented during periods of crisis in Canada. For this particular initiative, the crisis was the COVID-19 pandemic, but it could have been different one. As a fundamental transferability lesson, this article shows that when the regular functioning of clinical services is severely impaired by a crisis of whatever form, certain strategies can be tried in innovative ways according to the needs of the population and the unique context and resources of each country.

This project was conducted in Montreal in the province of Quebec in Canada. This context obviously makes this case-study unique, yet we believe its lessons can be transferred to other countries. During the pandemic, cases of COVID-19 were high in Canada, but its strong publicly-funded health care system responded, perhaps not perfectly, in a well-coordinated way. The possibility of including a peer support worker within a clinical team during this pandemic period could have been different if the healthcare system was simply overwhelmed and on the verge of collapse. Also, the structure of the Canadian health care system offers access to care for certain people who may not have a proof of coverage, including some people experiencing homelessness. Finally, in the province of Quebec, the Ministry of Health also includes social services (even in its official name) that make the inclusion of peer support workers in a clinical context somehow more acceptable, given their unique bridging role between the medical and social worlds.

The implementation trajectory of this project shows that it is possible to integrate people with a lived experience of homelessness within a team offering professional services (both medical and social) to people experiencing homelessness, even when the context of integration does not appear favorable at first. During a period of crisis, although it could be considered common sense for clinical teams to protect and reduce existing services rather than expand them by implementing novel initiatives, this project has shown that crises can serve as incubators for such initiatives.

Therefore, the concept of *crisis* needs to be reexamined by clinical and research teams as not only disturbing but also enabling. Thus, a particular crisis context can help clear the way forward for fostering relationships between the research and clinical worlds to collaborate on important issues such as homelessness. With the right mindset, crises can force us all to nurture the small, yet multiple and important relationships, slowly but surely, at the speed of trust.

## Key elements

1. Crises can precipitate numerous challenges but also unique opportunities for the implementation of peer support initiatives.
2. Implementing a peer support initiative in homelessness during a period of crisis goes beyond the application of implementation theories, since it also requires the materialization of concepts, principles, and values into people, relationships, and tangible practices.
3. Research teams can serve as external assets for clinical teams during a period of crisis, providing an “opportunity to breathe, innovate, and adapt” while clinicians focus on delivering services.
4. Even during a period of crisis, certain key stages cannot be skipped when the goal is a successful implementation of a peer support initiative:
  - Particularly, the initial planification and design of a peer support initiative requires time, but also allows for the choice of an adequate clinical hosting site that may increase the likelihood of a conducive “fit” between the participating teams (research and clinical).
  - Having members of both clinical and research teams meeting together on a regular basis enables an easier collaboration between them and helps with building relationships.

## Footnotes

See Dumez and L’Espérance [44] for a classification of experiential knowledge applied to patients that also can be useful for illustrating the range of experiential knowledge of peer support workers.
